# Rhinovirus protease cleavage of nucleoporins: perspective on implications for airway remodeling

**DOI:** 10.3389/fmicb.2023.1321531

**Published:** 2024-01-05

**Authors:** Jennifer Moorhouse, Nicole Val, Shadi Shahriari, Michelle Nelson, Regan Ashby, Reena Ghildyal

**Affiliations:** Faculty of Science and Technology, University of Canberra, Canberra, ACT, Australia

**Keywords:** nucleoporin (Nup) 153, rhinovirus, airway remodeling, asthma, transdifferentiation

## Abstract

Human Rhinoviruses (RV) are a major cause of common colds and infections in early childhood and can lead to subsequent development of asthma via an as yet unknown mechanism. Asthma is a chronic inflammatory pulmonary disease characterized by significant airway remodeling. A key component of airway remodeling is the transdifferentiation of airway epithelial and fibroblast cells into cells with a more contractile phenotype. Interestingly, transforming growth factor-beta (TGF-β), a well characterized inducer of transdifferentiation, is significantly higher in airways of asthmatics compared to non-asthmatics. RV infection induces TGF-β signaling, at the same time nucleoporins (Nups), including Nup153, are cleaved by RV proteases disrupting nucleocytoplasmic transport. As Nup153 regulates nuclear export of SMAD2, a key intermediate in the TGF-β transdifferentiation pathway, its loss of function would result in nuclear retention of SMAD2 and dysregulated TGF-β signaling. We hypothesize that RV infection leads to increased nuclear SMAD2, resulting in sustained TGF-β induced gene expression, priming the airway for subsequent development of asthma. Our hypothesis brings together disparate studies on RV, asthma and Nup153 with the aim to prompt new research into the role of RV infection in development of asthma.

## 1 Introduction

Asthma is a chronic inflammatory lung disease characterized by episodes of bronchoconstriction (exacerbations or attacks) caused by many different stimuli, including virus infections (AIHW, 2023). Rhinovirus (RV) infections are the most common viral cause of exacerbations in children and adults with asthma (Nicholson et al., 1993; Johnston et al., 1995). Additionally, RV infection may predispose children to developing asthma (Brouard et al., 2016; Toivonen et al., 2016; Jartti and Gern, 2017). The thickened airway wall with increased mucus secretion characteristic of asthma is attributed to airway remodeling (AR). There appears to be an association of severe asthma with repeated RV infections. Persistent and repeated RV infections are observed in people with asthma (Kling et al., 2005; Zlateva et al., 2014), while repeated RV infections may lead to an injury-repair loop that induces AR. In this perspectives article, we hypothesize that RV induced disruption of nucleocytoplasmic transport results in sustained AR related gene expression, priming the airway for asthma development. This could be the mechanism whereby RV infection predisposes children to developing asthma.

## 2 Rhinovirus

The many strains of rhinovirus (RV) are the main etiologic cause of upper respiratory tract infections (URTIs), commonly referred to as the “common cold” (Pappas, 2018). The incidence of RV infections tends to decrease with increasing age, with infants and children experiencing an average of seven to 10 episodes of the common cold annually, while adults experience an average of only two to five episodes each year (Gwaltney et al., 1967; D'Alessio et al., 1976). In addition to causing approximately half of all URTIs, RV can cause asymptomatic infections (Makela et al., 1998; Juven et al., 2000) or, more seriously, severe lower respiratory tract infections (LRTIs), and exacerbations of asthma and chronic obstructive pulmonary disease (COPD) that may lead to death (see Supplementary Figure S1A; Mosser et al., 2002; Self et al., 2016; Kerr et al., 2021).

## 3 Asthma

Asthma is a chronic inflammatory pulmonary disease of the conducting airways, usually caused by an immunological reaction. Asthma causes episodes of bronchoconstriction, which result in coughing, wheezing, breathlessness, and tightness of the chest. These symptoms result from increased sensitivity of the airways, bronchial wall inflammation and increased mucus secretions (see Supplementary Figure S1B; Kumar et al., 2015). Airway narrowing is a classic phenotypic sign of asthma and can occur as a result of airway remodeling (AR; Carroll et al., 1993) discussed in the next section.

RV infection is the major cause of viral induced asthmatic attacks in adults and children (Mak et al., 2011). In a cross-sectional study of hospitalized children, 85% of asthma exacerbations were a result of respiratory viral infections, two thirds of which were caused by RV infection (Mak et al., 2011).

Some studies have shown links between RV infections in childhood and the development of asthma later in life (Brouard et al., 2016; Toivonen et al., 2016; Jartti et al., 2020). Exposure to RV during infancy can predispose children to asthma, potentially leading to the development of the condition (AIHW, 2023). Infants affected by RV-associated wheezing during the first 3 years of life experience a 10-fold increased risk of developing asthma by age six; with nearly 90% of the affected infants developing asthma (Jackson et al., 2008). Only 16% of children who were not affected by wheezing developed asthma by age six (Jackson et al., 2008).

## 4 Airway remodeling

AR refers to a series of physical and structural changes to the airway wall that increases wall thickness and reduces the passage of air through the airway as shown in Supplementary Figures S1B,C (Breton et al., 2018; Michalik et al., 2018). These structural changes are indicative of repetitive airway injuries and are found in nearly all asthmatic airways (Elias et al., 1999; Breton et al., 2018; Hsieh et al., 2023). The result of these injuries can include subepithelial fibrosis, increased smooth muscle mass (thickening), gland enlargement, neovascularization and epithelial alterations as shown in Supplementary Figures S1B,C (Bergeron et al., 2010). One of the main structural changes observed during AR includes the transdifferentiation of epithelial cells to mesenchymal cells (EMT) and fibroblasts into myofibroblasts (FMT). Since myofibroblasts are practically absent in normal airways, FMT is one of the key events contributing to the chronic sequelae of asthma (Hackett et al., 2009), ultimately leading to permanently impaired pulmonary function (Pascual and Peters, 2005; Breton et al., 2018; Michalik et al., 2018). FMT can occur as part of a normal response to injury and, when no longer required, myofibroblasts undergo apoptosis or transition back into fibroblasts (Breton et al., 2018; Michalik et al., 2018). However, this does not appear to be the case in asthmatic airways (AA) where myofibroblasts remain after their initial purpose has finished, contributing to AR and chronic impairment (Breton et al., 2018; Michalik et al., 2018). Transforming Growth Factor-β (TGF-β) is a well characterized profibrotic cytokine that is elevated in the asthmatic lung. Importantly, TGF-β is a major inducer of both EMT and FMT (Breton et al., 2018; Walker et al., 2019) and a key contributor to AR.

## 5 TGF-β

TGF-β is part of a family of growth factors responsible for cell proliferation, tissue regulation, differentiation, and apoptosis (Kubiczkova et al., 2012; Walton et al., 2017). A ubiquitously expressed, secreted cytokine, TGF-β plays important roles in many physiological and pathological processes during development and in carcinogenesis (Chaudhury and Howe, 2009; Baba et al., 2022). TGF-β is induced in response to a variety of stimuli including RV infections (Dosanjh, 2006; Xia et al., 2017; Wieczfinska et al., 2022). TGF-β was not induced when primary bronchial epithelial cells were infected with low levels of RV (Bedke et al., 2012) and the authors concluded that basal endogenous production of TGF-β contributed to the observed effect on RV infection in cells from asthmatic airways. A recent study found upregulation of TGF-βR (TGF-β receptor) activity in RV infection *in vitro* and *in vivo*, implying increased TGF-β production (Dy et al., 2023).

**Figure 1:**
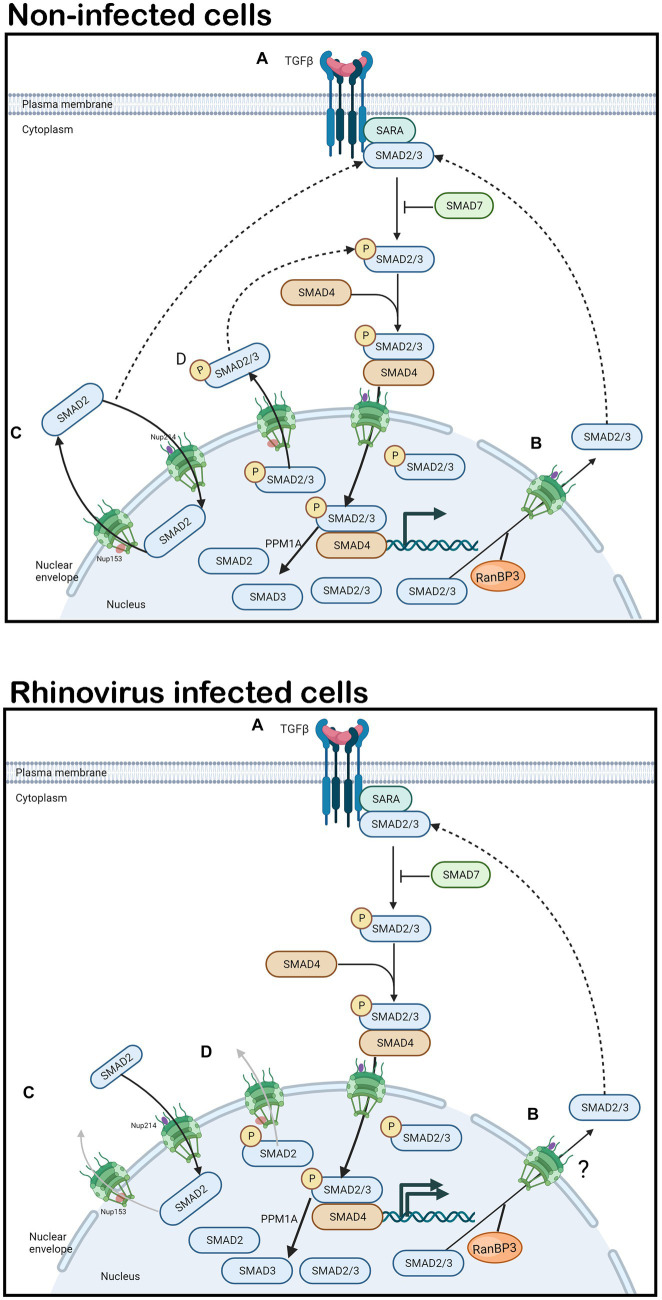
TGF-β1 signaling and SMAD2 shuttling in non-infected and rhinovirus infected cells. In non-infected or rhinovirus infected cells **(A)** TGF-β1 binds to the constitutively phosphorylated receptor TβRII. TβRII and TβRI receptors dimerize, the TβRI receptor becomes phosphorylated. Activated TβRI in turn phosphorylates SMAD2/3. SMAD7 can inhibit SMAD2/3 phosphorylation, thereby terminating TGF-β1 signaling. SMAD2/3 heterodimerize and bind to SMAD4, forming a cytosolic complex that translocates to the nucleus in an importin-dependent manner where it initiates airway remodeling associated gene expression. PPM1A acts to dephosphorylate SMAD2/3. **(B)** RanBP3 mediates nuclear export of dephosphorylated SMAD2/3 in a CRM1 independent manner that is not fully elucidated. **(C,D)** SMAD2 is continually imported into the nucleus by binding to Nup214 and exported by binding to Nup153, in stimulated and unstimulated cells in non-infected cells. In rhinovirus infected cells, Nup153 is cleaved, leading to inhibition of SMAD2 shuttling. SMAD2 and its phosphorylated form accumulate in the nucleus resulting in continued gene expression. Solid arrows denote signaling/transport direction, dotted arrows denote feedback mechanisms, faded arrows denote inhibition.

TGF-β signals through two receptor classes (Finnson et al., 2008; Miller and Hill, 2016) resulting in signaling cascades dependent on SMAD proteins. SMADs are intracellular transcription factors and key intermediates in TGF-β signaling (Bedke et al., 2012; Miller and Hill, 2016). A well-studied pathway associated with fibrosis is the TGF-β1/Activin receptor like kinase 5 (ALK5) pathway (Figure 1A, image labeled “non-infected cells”), which transduces intracellular signals through SMAD2/3 in most cell types (Finnson et al., 2008). Upstream receptor dependent interactions result in the phosphorylation of SMAD2/3, promoting their binding to SMAD4 to form a cytosolic complex (Kamato et al., 2013). The SMAD2/3/4 complex translocates to the nucleus where transcription of target genes is activated or repressed (Xu et al., 2012) inducing fibrosis (Walton et al., 2017). For details of the signaling pathway please refer to excellent published reviews (Kubiczkova et al., 2012; Massague and Sheppard, 2023). SMAD2/3 subsequently become dephosphorylated and Ran-binding protein 3 (RanBP3) exports them back to the cytoplasm for recycling or termination of TGF-β1 signaling (Dai et al., 2009; Figure 1B, image labeled “non-infected cells”).

## 6 Nucleocytoplasmic transport of SMAD2/3

SMAD2/3 and SMAD4 continuously shuttle between the cytoplasm and the nucleus in unstimulated as well as stimulated cells, providing a dynamic pool that is competitively drawn by cytoplasmic and nuclear signal transduction partners. While nuclear export of SMAD4 is dependent on the nuclear exporter CRM1, SMAD2/3 are exported via CRM1 independent mechanisms (Inman et al., 2002). SMAD3 is exported into the cytoplasm by Exportin 4 in a Ran GTPase dependent manner (Kurisaki et al., 2006). In unstimulated cells, SMAD2 is imported into the nucleus by its direct interaction with CAN/Nup (Nucleoporin) 214; it is exported to the cytoplasm in stimulated and unstimulated cells by direct binding to Nup153 (Xu et al., 2002; Figures 1C,D, image labeled “non-infected cells”). Significantly, TGF-β receptor-mediated phosphorylation does not alter the affinity of SMAD2 for Nup153. SMAD shuttling during active signaling involves continuous (but low level) dephosphorylation. Importantly, dominant-negative CAN/Nup214 or Nup153 constructs interfere with TGF-β activation of SMAD-dependent transcription. Exactly how or even if RanBP3 and Nup153 dependent nuclear export of SMAD2 synergize, compete, or compensate for each other is not clear. Cleavage of Nup153 and the subsequent nuclear accumulation of SMAD2 (Figure 1, image labeled “rhinovirus-infected cells”) could result in continual TGF-β stimulation as is observed when SMAD2 interaction with RanBP3 is inhibited (Dai et al., 2009).

## 7 RV, NUP153, and nucleocytoplasmic transport

The RV proteases 2A and 3C are responsible for the cleavage of several host proteins, in addition to their roles in proteolytic self-cleavage of RV polyprotein (Gustin, 2003; Amineva et al., 2004; Finnson et al., 2008; Castello et al., 2009; Caly et al., 2015; Jensen et al., 2015; Walker et al., 2016). RV proteases target the nuclear pore complex (NPC), cleaving several nucleoporins including Nup153, that make up the structure of the pore and enable transport through it with the result that nuclear transport in the infected cell is disrupted. The role of the NPC is to facilitate bidirectional nucleo-cytoplasmic shuttling of macromolecules through the nuclear membrane (Hoelz et al., 2011). While smaller molecules (<40 kDa) and ions can diffuse through the nuclear envelope freely, Nups, such as Nup153, are required to escort larger molecules (40-60 kDa) into and out of the nucleus in a cyclic fashion (Nofrini et al., 2016). Although 2A protease is capable of cleaving Nup153, 3C protease is thought to be the main protease responsible for cleavage in infected cells, as 3C protease activity correlates temporally with observed cleavage of Nup153 (Walker et al., 2013). The cleavage of Nup153 clearly contributes to the disruption of nuclear transport observed in infected cells (Gustin and Sarnow, 2002; Ghildyal et al., 2009).

TGF-β produced by RV infected cells could auto induce the signaling pathway resulting in nuclear import of SMAD2 (Figure 1A, image labeled “rhinovirus-infected cells”). In the context of cleaved Nup153, SMAD2/3 would not be able to be exported out of the nucleus (Figures 1C,D, image labeled “rhinovirus-infected cells”) resulting in continuous induction of TGF-β dependent genes, inducing AR. Indeed, a 2017 study by Minor and Proud found that RV or TGF-β alone caused 6 (+/− 3)% or 2 (+/− 1.1)% EMT in Beas-2B cells respectively, but together, resulted in 23.3 (+/− 7.6)% EMT (Minor and Proud, 2017).

Nup153 has been shown to be a key component in a variety of different processes which are independent of its role in transportation (Jacinto et al., 2015; Kitazawa and Rijli, 2017; Khan et al., 2020). While the exact mechanisms are not well defined, Nup153 is known to play a significant role in gene regulation and chromatin re-structuring (Kadota et al., 2020) by itself or in tandem with Sox2. Sox2 is a significant transcription factor responsible for a variety of regulatory processes in a range of cell types. The knock-down of either Sox2 or Nup153 results in significantly decreased levels of co-occupied genes in various models (Zhang and Cui, 2014; Kitazawa and Rijli, 2017; Kuo et al., 2020). Interestingly, Nup153 was the only nuclear structural protein enriched in a genome-wide analysis of 654 Sox2-enriched genes (Kitazawa and Rijli, 2017). Nup153 works with Sox2 to regulate cell type-specific transcriptional programs for the maintenance of neuronal progenitor cells and significantly, knock down of Nup153 leads to differentiation (Toda et al., 2017). An increasing number of studies show that Sox2 plays a vital role in EMT processes with TGF-β and Nup153 (Kuo et al., 2020).

The cleavage of Nup153 in RV infected cells could have significant impacts on gene expression directly, in addition to effects via nuclear retention of SMAD2.

## 8 Discussion

Previous work on RV biology from our group (Ghildyal et al., 2009; Walker et al., 2013, 2015, 2016; Caly et al., 2015; Jensen et al., 2015) and that from other groups (Gustin and Sarnow, 2002; Amineva et al., 2004; Castello et al., 2009; Watters and Palmenberg, 2011) has shown that RV proteases cleave several Nups, including Nup153, resulting in disruption of nucleocytoplasmic transport in infected cells. Our recent work on AR cell culture models (Breton et al., 2018; Walker et al., 2019) has demonstrated that increased TGF-β in the cellular milieu induces AR related pathways. Data from cell neuroscience (Zhang and Cui, 2014) and stem cell biology (Jacinto et al., 2015) has shown that Nup153 has an important role in regulation of transcription related to differentiation. We hypothesize that the cleavage of Nup153 in RV infection leads to accumulation of TGF-β induced SMAD2 in the nucleus and sustained AR associated gene expression, essentially priming the airway to increased risk of asthma in later years.

RV infection induces the production of TGF-β (Dosanjh, 2006; Xia et al., 2017; Wieczfinska et al., 2022), which binds to its cell surface receptors and induces an intracellular signaling cascade leading to nuclear localization of SMAD2/3/4. SMADs bind to specific gene loci and induce gene expression that drives AR. In uninfected cells, SMAD2 released from chromatin would translocate to the cytoplasm with the help of Nup153 (Xu et al., 2002) or RanBP3 (Dai et al., 2009). However, Nup153 is cleaved in RV infected cells (Walker et al., 2013), and we hypothesize that in that context, SMAD2 will be retained in the nucleus with consequent sustained AR associated signaling. We also hypothesize that the cleaved Nup153 is unable to continue its transcription functions in association with Sox2, further pushing the gene expression toward EMT/FMT and increased AR.

**Figure 2:**
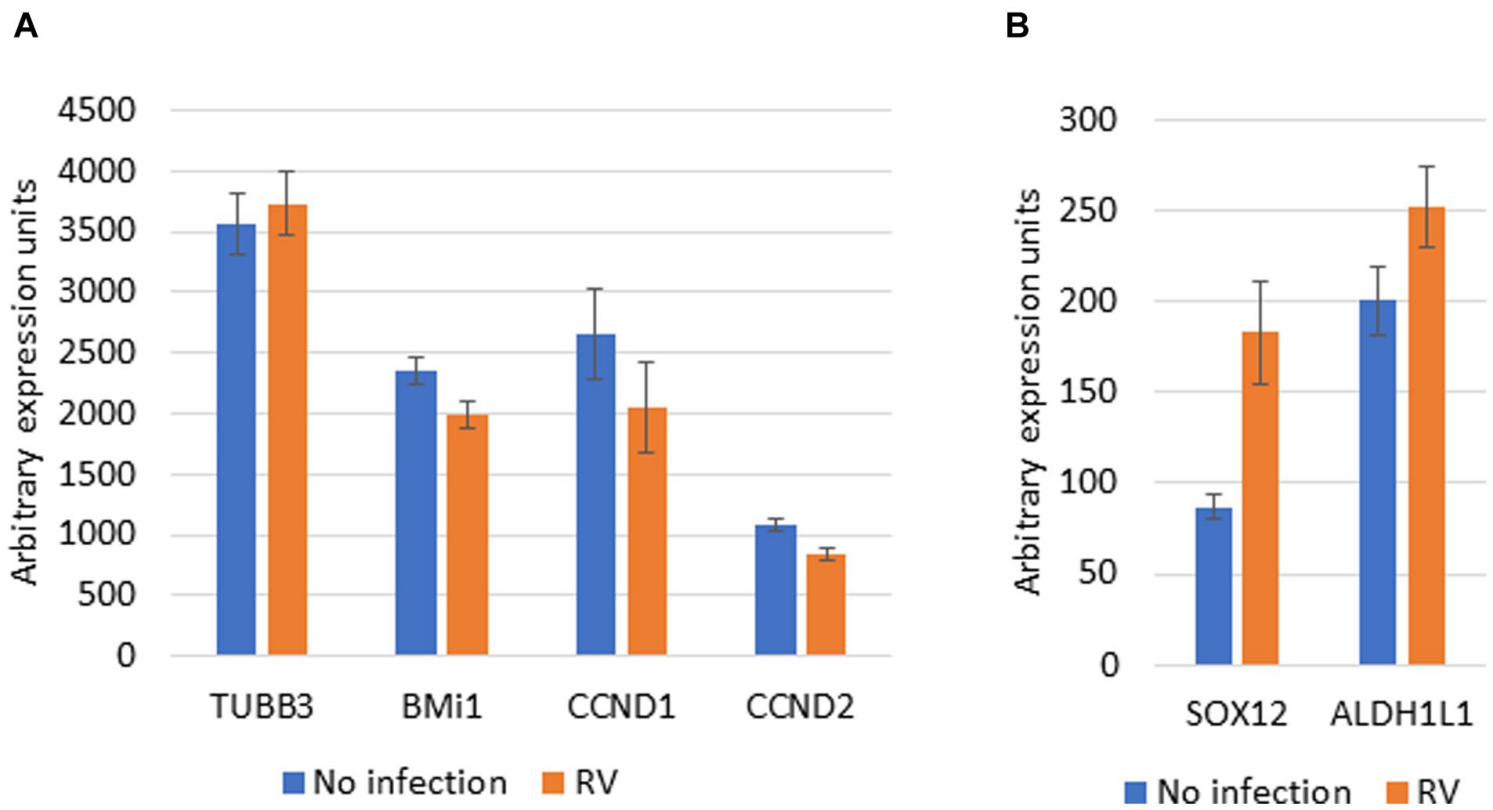
Effect of RV infection on Nup153 dependent genes. Microarray data for expression of Nup153 genes was downloaded from GEO Profiles Accension number GDS4832. Data on the gene expression levels (arbitrary units) for selected genes in presence and absence of infection with RV was extracted and is presented here. **(A)** Selected genes upregulated by Nup153. TUBB3, tubulin beta 3; BMi1, B cell-specific Moloney murine leukemia virus integration site 1; CCND1, cyclin D1; CCND2, cyclin D2. **(B)** Selected genes downregulated by Nup153. SOX12, SRY-Box Transcription Factor 12; ALDH1L1, Aldehyde Dehydrogenase 1 Family Member L1. The source data was generated by array expression profiling and used cells from four donors (Proud et al., 2012). Data are presented as Mean +/− SEM for data from all 4 donors.

If the above is true, RV infected cells should have decreased expression of Nup153/Sox2 associated genes that have a role in cellular differentiation. We performed a first such analysis on the GEO Profiles dataset GDS4832 that represents microarray expression profiling using cultured bronchial epithelial cells (four donors) after RV infection (Proud et al., 2012). TUBB3, SOX12 and ALDH1L1, that are downregulated by Nup153 (Toda et al., 2017), showed a trend for increased expression in RV infected samples (Figure 2A). BMi1, CCND1 and CCND2, that are upregulated by Nup153, showed a trend for decreased expression (Figure 2B). Although the changes are not statistically significant, this analysis provides preliminary support for our hypothesis. Future research could investigate the relationship between Nup153 function, TGF-β signaling and development of asthma in models where Nup153 is either downregulated or knocked out.

Our hypothesis predicts that RV infected cells will have increased levels of SMAD2 in the nucleus; this has not yet been tested. If our hypothesis holds true in the clinic, repeated RV infections would increase the risk of later development of asthma. Our hypothesis also predicts that allergen injury following on initial RV infection has increased risk of asthma development compared to allergen injury alone. Interrogation of large longitudinal clinical datasets should clarify these and other clinical predictions of our hypothesis.

## Data availability statement

Publicly available datasets were analyzed in this study. This data can be found at: GEO Profiles dataset GDS4832.

## Author contributions

JM: Conceptualization, Visualization, Writing – original draft, Writing – review & editing. NV: Visualization, Writing – original draft, Writing – review & editing. SS: Supervision, Writing – review & editing. MN: Writing – review & editing. RA: Methodology, Supervision, Visualization, Writing – review & editing. RG: Conceptualization, Supervision, Visualization, Writing – original draft, Writing – review & editing.
