# Supplementary figures and images for: Rhinovirus protease cleavage of nucleoporins: perspective on implications for airway remodeling
Source: Front Microbiol. 2024 Jan 5;14:1321531. doi: 10.3389/fmicb.2023.1321531 (PMC10797083; doi:10.3389/fmicb.2023.1321531)

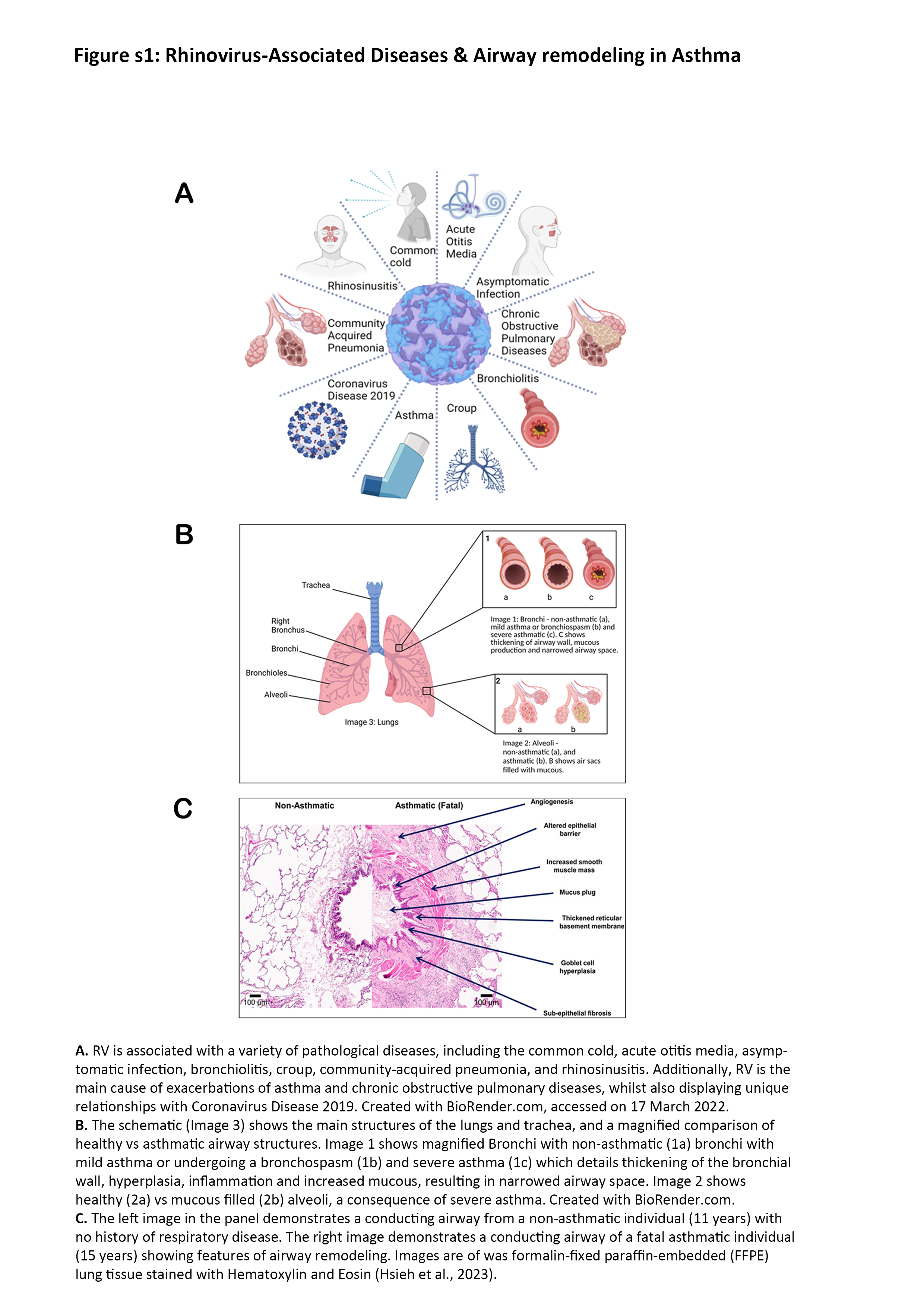

Supplement: Supplementary file 1 [file Image_1.TIF]
